# Infectious diseases surveillance in Pakistan: Challenges, efforts, and recommendations

**DOI:** 10.1016/j.amsu.2022.103838

**Published:** 2022-05-20

**Authors:** Wajeeha Bilal, Khulud Qamar, Samina Abbas, Amna Siddiqui, Mohammad Yasir Essar

**Affiliations:** a Faculty of Medicine, Dow Medical College, Dow University of Health Sciences, Pakistan; b Karachi Medical and Dental College, Pakistan; c Kabul University of Medical Sciences, Kabul, Afghanistan

**Keywords:** Infectious/communicable diseases, Public health, Disease surveillance systems, Pakistan

## Abstract

Public health remains a major concern in Pakistan, with communicable diseases including HIV/AIDS, hepatitis B, and C, and tuberculosis the leading cause of morbidity and mortality. Several factors contribute to the country's high risk of infectious disease epidemics, including overcrowded cities, unclean drinking water, inadequate sanitation, poor socioeconomic conditions, and low vaccination coverage. Due to the absence of a comprehensive surveillance strategy and mechanism, it has been difficult to manage infectious disease outbreaks effectively. The article offers insights into the various challenges faced by public and private healthcare sectors to control the spread of communicable diseases and proposes solutions to prevent crippling of the overburdened healthcare system on a national scale.

## 1 Introduction

A majority of infectious diseases are caused by bacteria, fungi, and viruses. These organisms are transmitted primarily through the following modes: contact, airborne, droplet, and vector [1]. Pakistan, being a developing country has a poverty rate of 30–40%, which is a significant factor that results in a high prevalence of infectious diseases such as malaria, hepatitis, HIV, dengue, tuberculosis, and influenza [2]. Additionally, causes of the widespread presence of infectious diseases include poor sanitation conditions, scarcity of basic health care resources, and lack of awareness of these diseases and their severity among the local population [2]. According to statistics, in 2020, 47,120 people suffered from dengue fever [2]. Moreover, 27,000 tuberculosis, 300,000 malaria, and 2.5 million Hepatitis B cases are reported annually [2]. Typhoid was diagnosed in 22,571 patients between 2016 and 2020, along with 8345 cases of measles presented in 2019 and 192 influenza patients presented in 2018 [2]. The incidence rate of HIV shows a pattern of increasing every year, being 0.12 for 2019 [3].

However, the emergence of COVID-19 has further burdened the already struggling health care system; there exists a similarity in the clinical manifestations of infectious diseases such as TB, Dengue, typhoid, and COVID-19 which include fever, cough, tiredness, and body aches [2,4]. This similarity in the route of infection and clinical symptoms also poses a risk for coinfection, which requires physicians to devise a treatment plan accordingly. This is only possible if adequate testing kits are present to diagnose patients and treatment resources are widely available [2].

In the previous years, no major actions have been performed by the government to combat infectious diseases. Nonetheless, widespread immunization of the population is a significant measure carried out by the government to eradicate infectious diseases [5]. Furthermore, the government took strict measures to limit the spread of COVID-19 since its emergence by imposing lockdowns and by making the following SOPs which include wearing masks, social distancing, and sanitization mandatory [6]. Strict surveillance has been performed to limit the spread of COVID-19, which can be implemented to control other infectious diseases in Pakistan [6].

## 2 Challenges and efforts

Pakistan, a developing country located in the sub-tropics, experiences seasonal outbreaks, causing an intense burden of infectious diseases (IDs) per year, placing the land among the heavily-ID burdened regions, declared by WHO [7]. On top of that, predisposing factors that are already known to cause infectious outbreaks are more prevalent in Pakistan, i.e., poverty, inadequate hygiene, increasing grounds for mosquito breeding, food insecurity, and lack of (or resistance to) vaccination. Notably i.e., cholera is the disease of poverty and inadequate hygiene. Therefore, despite being prone to infectious outbreaks, the state still lacks a proper surveillance system to manage IDs [8,9].Thus, it specifies the negligence of private and public health care standards in controlling the spread of IDs. Apart from negligence, the unaccountability, lack of follow-ups of existing infection control guidelines, and limited healthcare workforce with inadequate experience and knowledge in dealing with emerging outbreaks along with COVID-19 aggravate the spread of infectious outbreaks in the country.

Additionally, other factors that hamper the general upgrade of the state's healthcare include the lack of resources to reach an accurate differential diagnosis, demanding sufficient diagnostic kits, capacity, funds, and technical staff [2].Consequently, the co-occurrence of the epidemic of COVID-19 with other endemic infectious diseases in Pakistan is a source of double-burden on the state as it impacts the country's fragile health care system and profoundly complicates the diagnosis as discussed.

Hence, there must be running of infection control programs based on precautions, as elaborated by Yacob Habboush et al. and automated detection setup operating across the country to keep the spread of IDs in check [10]. Nevertheless, the risk assessment of IDs (vector-borne, air-borne, water-borne & food-borne) underscores the dire need for a real-time surveillance framework in Pakistan to efficiently cope with the imminent public health challenges due to the occurrence of IDs.

In Pakistan, a series of programs and projects have been launched to improve the health status of the population and to make communicable diseases less prevalent. In accordance with the 18th Constitutional Amendment, health services have been transferred to the provinces [11]. However, Pakistan Vision 2025, prepared in consultation with provinces, provides a road map that includes reducing the prevalence of infectious diseases, enhancing disease surveillance, and investing in primary and secondary health care [11]. In 2005, following the earthquake crisis, the Disease Early Warning System (DEWS) was set up as a way for health care workers to discover epidemic signs at an early stage in order to respond quickly and limit the impact of an outbreak [12]. This initiative aims to minimize morbidity and mortality associated with communicable diseases [12]. 40% of Pakistan's disease burden is caused by communicable diseases like malaria and tuberculosis [11]. In the public sector, the National Tuberculosis Control Programme (NTP) has achieved over 80% Directly Observed Treatment System (DOTS) coverage and served more than half a million TB patients in the past five years [11]. To reach the global target of 70% case detection, the program is steadily making progress [11]. A spike in vector-borne diseases like dengue fever in Punjab necessitated the development of a Disease Surveillance System (DSS), operational since July 2013 to prevent future outbreaks and coordinate action across the province [13]. Besides improving the availability of and quality of data, the system offers an early warning system and enables disease data to be correlated with geospatial mapping [13]. All levels of healthcare facilities in Punjab are collecting data for surveillance, and this data is stored on an HRS-centralised server, which includes programs such as Dengue Patients Surveillance, Polio Monitoring, Hepatitis Control, and TB Control [13]. The system currently tracks 26 communicable diseases listed by the WHO but is being expanded to track 81 diseases in total at the request of the Primary and Secondary Healthcare Department [13]. Using the surveillance system, health authorities were able to contain an outbreak of influenza late in 2015 and halt a measles epidemic in 2012 [13]. It also played a key role in controlling the dengue outbreak, resulting in a drop in cases from thousands in 2011 to less than 100 from 2012 onwards [13]. Khyber Pakhtunkhwa government also launched an Integrated Disease Surveillance System to determine health indicators and prevent outbreaks of diseases [14]. This 18-month pilot project launched in six districts is expected to be scaled up to the whole province, with all network members connected by SMS or the internet. Thus, rapid exchange of integrated information and the issuing of alerts regarding measles and diphtheria can be achieved to enable immediate response [14]. In partnership with Pakistan's National Institute of Health (NIH) as well as provincial and district level offices, CDC strengthens public health capacity and infrastructure, such as workforce development, hepatitis surveillance, and polio elimination [15]. A viral hepatitis surveillance system based in the four provinces and the capital of Pakistan has been operational since 2009, based on a partnership between the CDC and the Government of Pakistan [15]. USAID supports CDC's work in Sindh to implement and evaluate hepatitis prevention and control measures [15]. A project aimed at ensuring safe blood transfusions with the cooperation of the German Society for International Cooperation (GIZ) and the Kreditanstalt für Wiederaufbau (KfW) has been implemented in all four provinces, which will contribute to addressing the hepatitis issue in the country [15]. Through a cooperative agreement with the Pakistan National Institute for Health since 2004, CDC has also been supporting influenza surveillance in Pakistan [15]. A network of five geographically representative surveillance sites has been set up to conduct surveillance for influenza-like illnesses in outpatient settings [15]. Thus, Pakistan's health system has established a large infrastructure of primary and secondary health facilities in rural and urban areas to ensure timely provision of better health facilities (Table 1).

**Table 1:** Disease surveillance programs operating across the country.

Surveillance Systems and Programs	Functionality
Pakistan Vision 2025 [11]	It includes strategies aimed at reducing infectious diseases, improving disease surveillance, and investing in primary and secondary health care.
Disease Early Warning System (DEWS) [12]	By detecting epidemic signs at an early stage, this initiative aims to reduce morbidity and mortality related to communicable diseases by responding quickly and minimizing the impacts of an outbreak.
National Tuberculosis Control Programme (NTP) [11]	Using a comprehensive approach to TB, this program seeks to control, prevent, and eventually eradicate the disease from Pakistan.
Disease Surveillance System (DSS), Punjab [13]	The goal is to reduce the incidence of outbreaks of vector-borne diseases and coordinate action across the province.
Integrated Disease Surveillance System, Khyber Pakhtunkhwa [14]	Aimed at determining health indicators and preventing epidemic outbreaks, this system is launched in six districts.
Centers for Disease Control and Prevention (CDC) in collaboration with Pakistan's National Institute of Health (NIH) [15]	Working with several key public health institutions in Pakistan, it strengthens capacity and infrastructure in order to address important public health issues such as workforce development, hepatitis and influenza surveillance, and polio eradication.

## 3 Recommendations and conclusion

The country's current status requires alert and prompt actions to control the spread of communicable diseases among urban and rural areas. To sort out the out-of-hand situation, studies accentuate that the government and media must integrate awareness for the requirement of hygiene, the primary symptoms of diseases, and the need for early diagnosis to prevent worsening of health and spread of infection [16].Specific disease transmission can be controlled by specific management methods. Cholera can be reduced with clean water availability, via filtration or the use of chlorine tablets that kill bacteria. Typhoid can be controlled by spreading awareness for utilizing boiled water for drinking and avoiding ingestion of contaminated food. Completely cooked meat and eggs will kill the causative organism. HIV, AIDS, and Hepatitis are diseases that are commonly transmitted by non-sterile syringe usage, unprotected sexual intercourse, breastfeeding, pregnancy from mother to child, or any kind of unprotected contact with bodily fluids. Enforcement for proper cleanups at public places like toilets, stations, waiting rooms, offices, hospitals must be taken into action [17].Onset of COVID-19 has given a head start for the public on taking hygiene seriously especially by washing hands as sanitization prevents infectious organisms from invading into areas like the nose, mouth, and hands. Use of sanitizers, maintaining quarantine after traveling, avoiding people to the crowd at areas, and wearing medical face masks reduces the chances for contracting not only COVID-19 rather also other viral respiratory diseases including adenoviruses, influenza, respiratory syncytial virus, metapneumovirus, parainfluenza virus, rhinovirus enterovirus, coronavirus, coronaviruses parainfluenza viruses, severe acute respiratory syndrome-associated coronavirus, adenoviruses, and human bocavirus, coxsackie/echoviruses [18]. Institutes, schools, colleges, workplaces, and public places can commend the practices to remain even after COVID-19 diminishes. Free healthcare camps and diagnostic facilities should be provided for those unable to afford checkups. Hospitals of Pakistan have been in the past and will in the future continue to send healthcare workers and trained volunteers to visit rural areas for diagnostic and treatment purposes. For infected persons, immediate treatment and quarantine can be taught to all communities through social media awareness on using over-the-counter medication for fever, flu, chills, diarrhea, and nausea and for non-infected persons, importance of sanitization is the requirement to isolate from unwell persons. As a preventive measure recognition for the hallmark symptoms for various diseases must be highlighted amongst the higher, middle, and lower socioeconomic classes of Pakistan [19]. This all can be carried out by assigning healthcare workers a group of laymen they can educate on the transmission, clinical symptoms, treatment, and prevention of all non-communicable diseases, later the audience has to follow up with a test that ensures their complete understanding of the disease. These recommendations provide feasible means to control and manage non-communicable diseases efficiently if taken into action by medical and non-medical individuals. This calls for a unifying effort from all communities of all classes.

Partnering with COVID-19 the infectious diseases that are cholera, malaria, HIV, Hepatitis, typhoid has shown an increased prevalence in the following years. This is mainly due to reasons such as overpopulation, frequent contact with disease carriers, lack of sanitization, lack of hygiene, inaccessibility to treatment due to unaffordability, lack of medical facilities, inefficient treatment as health care workers is not skillfully trained, self-diagnosis, and intake of wrong medication all of which have worsened the health of infected individuals. Resulting in high death rates, efforts have been made in Pakistan to manage and prevent diseases from having a deteriorating effect by Ministry of health and several NGOs. To improve the standard of health care and have an effective disease control, many measures like educational awareness and financial support should be provided by authorities. As well as combined effort in handling the disease within a community regarding its transmission, infection, clinical symptoms, treatment, and prevention of diseases.

## Ethical approval

N/A.

## Source of funding

None.

## Authors’ contributions

Mohammad Yasir Essar conceived the idea and design, Wajeeha Bilal wrote the efforts, abstract, edited the revised draft and organized references; Samina Abbas wrote the introduction, Amna Siddiqui wrote the challenges, and Khulud Qamar wrote recommendations and conclusion; Mohammad Yasir Essar made the critical comments and revision. All authors revised and approved the final draft.

## Trial registry number

1. Name of the registry: N/A.
2. Unique Identifying number or registration ID: N/A.
3. Hyperlink to your specific registration (must be publicly accessible and will be checked): N/A

## Guarantor

N/A.

## Consent

N/A.

## Declaration of competing interest

None.
